# Comparative Evaluation of AI Chatbots for Medical Research: Cross-Sectional Observational Study

**DOI:** 10.2196/103592

**Published:** 2026-11-03

**Authors:** Francesco De Caro, Federico Della Rocca, Alessandra Mea, Matteo Tomeo, Simona Caruccio, Myriam Baldi, Giuseppe D'Arienzo, Raffaele De Caro, Emanuela Santoro, Giuseppina Cersosimo, Giovanni Boccia, Mario Capunzo, Giuseppina Moccia

**Affiliations:** 1 Public Health Laboratory for the Analysis of Community Health Needs, Department of Medicine and Surgery, University of Salerno, Baronissi, Campania, Italy; 2 Department of Medicine and Surgery, University of Salerno, Baronissi Campus, Baronissi, Campania, Italy, 39 3385430632; 3 Department of Political and Social Studies, University of Salerno, Fisciano, Campania, Italy

**Keywords:** AI chatbots, hallucinations, bibliographic references, medical research, ChatGPT, Grok, Meta AI, Perplexity

## Abstract

**Background:**

The use of generative AI in research and clinical practice is rapidly expanding, offering unprecedented capabilities in pattern extraction and data analysis. However, its application raises critical concerns regarding the accuracy and reliability of the information provided.

**Objective:**

The aim of this study is to analyze and compare the performance of 4 generative AI platforms (ChatGPT, Grok, Meta AI, and Perplexity) in retrieving bibliographic references within the medical and health care field and to assess factual accuracy and information retrieval strategies.

**Methods:**

A cross-sectional observational study was conducted based on the simulation of 3 bibliographic queries with varying levels of specificity (aflatoxin toxicity, hospital sanitization, and human papillomavirus vaccination). Each prompt was administered in triple iteration on each platform operating in “Guest” mode. Factual accuracy was verified through a manual cross-check against the Scopus and Google Scholar databases using a hierarchical query strategy prioritized by DOI. The incidence of hallucinations was quantified via a standardized scoring system (range 0‐5) based on metadata correctness. Categorical associations were analyzed using the Pearson chi-square test, while nonparametric continuous variables (hallucination score, publication year, and citation count) were compared using the Kruskal-Wallis test.

**Results:**

Of the 360 requested references, 273 were generated (task completion rate: 75.8%). Among these, 190 adhered to the requested article format (instruction adherence rate: 69.6% of generated outputs). Fact-checking revealed that 128 of these 190 validly formatted articles (metadata accuracy rate: 67.4%) showed an exact match with real evidence, resulting in an overall strict yield of 35.6% (128/360) from the initial requested references. The ability to provide real references depended significantly on the AI model used (χ_6_²=192.5; *P*<.001). Kruskal-Wallis tests revealed statistically significant differences among the models across all analyzed dimensions (*P*<.001). Grok emerged as the most reliable model, achieving 71 out of 90 (78.9%) exact matches, exhibiting minimal hallucinations (mean score 0.2, SD 0.5), and showing a marked propensity toward recent sources (median year 2022, IQR 2019.0-2024.8). ChatGPT demonstrated robust stability, with 46 out of 90 (51.1%) exact matches, and anchored itself to older (median year 2020, IQR 2011.0-2023.0), highly cited literature (median 73.5, IQR 10.8‐718.0 citations). Perplexity exhibited critical rates of total hallucination and a structural inability to distinguish scientific articles from gray literature. Meta AI systematically failed the task, producing entirely incorrect data on a single occasion (0/10, 0% exact matches).

**Conclusions:**

Under the specific testing conditions of this study, the evaluated large language models demonstrated a lack of the necessary reliability to replace validated bibliographic databases, primarily due to response instability and a high incidence of metadata fabrication. The results highlight the urgency of introducing structured AI literacy programs across medical education, promoting the use of such technologies exclusively as a complementary tool and always subject to rigorous human fact-checking.

## Introduction

### The Context: The Age of AI

The use of AI now offers the possibility to examine and extract patterns from large and highly complex datasets. While AI can ensure unprecedented speed in specific tasks such as data mining, predictive analytics, and pattern recognition, its accuracy remains highly variable and dependent on the specific application [1].

Over the past decade, the ongoing evolution of AI has profoundly impacted many aspects of society and clinical practice [2]. By leveraging vast amounts of data, AI provides an essential wealth of resources for research and public health, making medical and scientific information much more accessible. The integration of these technologies into the educational sector has ushered in a new era of technology-supported learning, creating highly innovative prospects for health care education as well [3].

In this context, the integration of natural language processing models is proving to be a game-changer for radically improving the accessibility and management of information in health care. Specifically, large language models (LLMs) have established themselves as cutting-edge tools, endowing machines with the ability to understand, process, and generate text in a way that is almost indistinguishable from human communication [4].

The medical and health care sector represents one of the most promising domains for the application of LLMs. In recent years, these models have garnered significant attention due to their ability to outperform traditional computational approaches in specific natural language processing tasks, such as medical text summarization, conversational triage, and translation [5,6]. However, high performance in these semantic and generative tasks cannot be indiscriminately generalized to domains requiring strict factual retrieval, such as academic research and evidence-based medicine (EBM).

By analyzing massive amounts of unstructured text—from medical records to academic articles—LLMs prove essential in supporting decision-making processes. They assist health care professionals in diagnosis, treatment recommendations, and interpretation of complex cases and reports and even play a key role in patient education and in identifying emerging trends in scientific research [4,5].

However, despite their remarkable potential, the adoption of LLMs in medicine raises several concerns that require careful evaluation by health care professionals and researchers [6].

Research has highlighted that such systems exhibit significant structural and epistemological limitations: they can inherit biases stemming from their training datasets or algorithmic optimization processes. A particularly critical issue in the clinical field is represented by “hallucinations”—the generation of information entirely devoid of factual basis—a phenomenon that tends to be exacerbated when addressing novel, complex, or cutting-edge topics [7].

In the psychological domain, outside the realm of AI, the term “hallucination” refers to a specific perceptual phenomenon consisting of a sensory illusion devoid of any basis in reality, which the subject perceives as authentic. The anomaly wherein AI systems produce inconsistent or erroneous outputs bears striking similarities to the clinical definition of the term, thus justifying this linguistic borrowing. Indeed, “hallucinated” text appears fluent and natural, thereby masking its inherent inaccuracy. Although seemingly logically anchored to the user-provided context, it is actually rooted in fictitious or objectively unverifiable premises. Consequently, within the field of AI, the current standard defines a hallucination as the generation of content that is either baseless or incongruent with the original source material [8].

It should be noted, however, that not all experts agree with the use of this terminology. Indeed, some authors contend that it would be more appropriate to speak of “fabrications and falsifications” rather than hallucinations: a fundamental distinction aimed at avoiding the anthropomorphization of a machine that is merely “inventing” nonexistent data [9]. For the purposes of this paper, the term “hallucination” will conventionally be adopted to identify this phenomenon, in alignment with the prevailing nomenclature in the current technical discourse.

AI-generated hallucinations can originate from multiple interconnected factors, including the reliance on nonspecialized or biased training datasets, the inherently probabilistic nature of text generation, the lack of adequate contextual parameters in user prompts, and the structural limitations of language models in sequential and deductive reasoning [10].

Language models are not inherently designed as academic search engines or relational databases: they operate as “probabilistic engines” trained to generate fluent and plausible text, not to retrieve verified data. Their tendency to “fabricate” seemingly flawless article titles, attributing them to real authors and existing journals, despite being entirely nonexistent in the published literature, is a widely documented phenomenon that threatens the very integrity of the scientific method. While in purely creative or brainstorming contexts, algorithmic hallucinations may be considered a minor flaw, in the field of scientific research and evidence-based clinical practice (EBM), they represent a critical risk [11,12].

Recent studies have underscored the need to critically evaluate the accuracy and reliability of chatbot-generated responses to direct medical queries, emphasizing how the integration of such tools into daily practice requires rigorous validation protocols to ensure their safe and effective deployment [13].

While the phenomenon of AI-generated hallucinations in medical content is increasingly documented, prior research presents significant methodological limitations, particularly concerning prompt design. For instance, recent efforts to quantify AI reliability, such as the reference hallucination score (RHS) proposed by Aljamaan et al [14], have relied on highly structured, engineered prompts. In such studies, the models are explicitly instructed to output predefined metadata fields (eg, authors, title, journal, and DOI). Although valuable for assessing theoretical capabilities under optimal conditions, this heavily guided approach does not reflect the real-world interactions of everyday users—such as students, health care professionals, or laypersons—who typically formulate open, unstructured queries.

Furthermore, earlier studies [11,12] have predominantly focused on single-model evaluations, mostly testing initial versions of ChatGPT, lacking a comparative framework across different algorithmic architectures.

These gaps leave a critical question unanswered: how vulnerable is a standard, unguided user to bibliographic hallucinations when interacting with the newest generation of LLMs? Our study directly addresses this gap by employing an open-ended prompt design. By deliberately omitting instructions on how to structure the output, this study evaluates not only the factual accuracy of the models but also their autonomous capacity to recognize, retrieve, and correctly format scientific evidence without explicit guardrails. This approach provides a more realistic and rigorous assessment of the comparative reliability of newer platforms—such as Grok, Meta AI, and Perplexity—in the context of specialized public health and clinical research.

### Aims of the Study

In light of critical issues highlighted in the literature, the primary objective of this study is to evaluate and compare the outputs generated by different AI platforms in response to specific bibliographic research questions in the medical and health care field. The investigation was designed to simulate typical queries from a broad spectrum of users—ranging from health care professionals to students and even laypersons with no medical background—while subsequently subjecting the obtained results to rigorous methodological validation conducted by domain experts.

Specifically, the research aims to measure the precision and accuracy of the references provided, verifying the exactness of metadata and the actual existence of the proposed articles, in order to quantify the reliability of the models and to highlight the presence of any hallucinations. Beyond factual validation, the study intends to explore the underlying logical and selection mechanisms intrinsically used by the models to compile reference lists. Therefore, it seeks to analyze which parameters guide AI in choosing the retrieved articles, evaluating key factors such as thematic coherence with the user’s query, scientific impact (inferred from citation counts), and the recency of the sources (publication year).

Through this analysis, the study aims to provide a comprehensive overview not only of the accuracy limitations of various chatbots but also of the information retrieval strategies driving their responses, offering valuable insights for their informed use across clinical, academic, and everyday settings.

## Methods

### Study Design and Selection of Tools

To assess the effectiveness and reliability of AI in the health care sector, a cross-sectional observational study was conducted.

Four of the most widespread and currently used generative AI platforms have been selected for analysis: ChatGPT [15], Grok [16], Meta AI [17], and Perplexity [18].

The selection of these tools was based on the following criteria: a free-of-charge service, open and unrestricted access, and usability without requiring user account registration.

These inclusion criteria were adopted to simulate the conditions of a standard user seeking immediate access to medical information. The use of “Guest” mode was implemented to mitigate user-history bias and prevent influence by preexisting account cookies, although we acknowledge this does not guarantee absolute algorithmic neutrality, as IP-based geolocation and intrinsic model biases remain active.

### Query Formulation

In order to assess the bibliographic search capabilities of AI systems, 3 health-related queries were formulated. These were structured to provide a variable gradient of specificity, ranging from highly specialized topics to broader public health themes.

To ensure linguistic uniformity and isolate the topic investigated as the only independent variable of the experiment, the syntactic structure of the questions was kept constant.

The three prompts, administered in Italian, were as follows:

1. D1 (specific): “Conduct a literature review, citing 10 articles dealing with the toxicity of aflatoxins.”
2. D2 (intermediate): “Conduct a literature review, citing 10 articles dealing with the sanitization of hospital environments.”
3. D3 (General): “Conduct a literature review, citing 10 articles dealing with human papilloma virus vaccination.”

### Data Collection

In order to standardize the operational conditions and mitigate technical biases, all queries were performed using a single personal computer and the Google Chrome web browser in “Guest” mode.

All tests were conducted from the geographical region of Italy, with the browser interface language set to Italian. The entire prompt administration phase was completed within a strict 24-hour time window (March 24‐25, 2026). This rigorous methodological precaution was adopted to minimize confounding variables associated with the continuous updates of the underlying LLMs and the machine learning evolution of the AIs over time.

The following free versions, accessible without user authentication (Guest mode), were used: ChatGPT (defaulting to the GPT-4o architecture available for unauthenticated users), Grok (Grok-2 architecture family), Meta AI (powered by the Llama 3 model series), and Perplexity (standard free search model).

The querying protocol was executed in accordance with these standardized steps for each AI and each question.

1. Initialization: The browser was opened from scratch in “Guest” mode. A standardized procedural exception was required for accessing the Meta AI platform: since the system required age verification upon opening the link (via the message “Select your date of birth to confirm you can use Meta AI”), it was decided to invariably enter the year 1900. This choice, corresponding to the earliest historical option selectable in the drop-down menu, was adopted to neutralize the demographic variable and avoid any algorithmic bias related to user age profiling.
2. Administration: The predefined prompt was pasted directly into the AI’s input bar.
3. Data acquisition: The generated output (the AI’s response containing the bibliographic references) was fully acquired and archived simultaneously in 3 digital formats: image format with screenshot of the screen (.jpg), document format (.pdf), and text format (.txt).
4. Closure: Upon completing the data saving process for every single iteration, the browser was completely closed.
5. Iteration repetition: To assess the reproducibility and internal consistency of the models, the entire administration procedure for each query was repeated three consecutive and identical times for each AI platform. Prior to each iteration, a new browser session was initiated in “Guest” mode. The implementation of the reset procedure was a deliberate and methodical process, intended to ensure the integrity and objectivity of the algorithmic responses. This procedure was designed to prevent any potential biases or influences from previous search history, cookies, or session memory, thereby maintaining the accuracy and fairness of the algorithmic responses. The responses obtained during the 3 iterations were classified and labeled in chronological order as R1, R2, and R3.

In the early stages, the phase of data acquisition involved five AI platforms (ChatGPT [15], Grok [16], Meta AI [17], Perplexity [18], and OpenEvidence [19]). However, during prompt administration, the use of OpenEvidence revealed operational limitations related to the platform’s policies: “guest” access (without registration or login) only allowed the generation of a response for the first query (D1), blocking subsequent queries. In order to guarantee the strict homogeneity and statistical comparability of the study sample across all prompts (D1, D2, and D3), it was decided to exclude OpenEvidence from the analysis.

### Data Extraction

All textual data generated by the AI models were extracted and structured into a dedicated tabular database created using Microsoft Excel software. Based on the bibliographic references provided by the AIs—accounting for 4 platforms, 3 queries (D1, D2, and D3), 3 iterations per query (R1, R2, and R3), and 10 articles requested per iteration—an initial theoretical total of 360 items was collected for the subsequent evaluation phase (4 AIs × 3 prompts × 3 iterations × 10 requested articles).

A rigorous eligibility criterion regarding the source type was applied to these 360 references, based on strict adherence to the literal wording of the administered prompt. In accordance with the explicit stipulations of the prompt, which called for the citation of “10 articles,” only responses containing authentic scientific articles were considered valid and extracted for further analysis. Any other type of source suggested by the AI (including books, manuals, guidelines, institutional reports, or general websites) was classified as “unacceptable” and excluded from the final dataset.

### Data Analysis

The bibliographic references that were deemed valid following the extraction process were then subjected to a 3-stage analytical process. The purpose of this process was to determine the formal completeness and factual accuracy of the generated outputs.

#### Phase 1: Evaluation of Output Completeness

For each article considered, the formal completeness of the AI-generated response was assessed. The text generated was checked for the explicit presence of the following bibliographic elements, with each occurrence recorded using a dichotomous indicator (presence/absence): title, authors, journal or publication source, year of publication, DOI, link to the article, and comment or brief summary of the article

#### Phase 2: Data Verification and Comparison

The second phase involved a manual cross-check to verify the existence of the cited articles and the accuracy of the provided metadata.

To strictly establish the verifiable “real evidence” as our gold standard, researchers applied a hierarchical query strategy: the primary search was conducted using the DOI provided by the AI model. If the DOI was absent, invalid, or led to a nonexistent link, a secondary targeted search was performed using the exact article title combined with the authors’ names.

The manual cross-check was conducted independently by 3 researchers. The assessors were blinded to the identity of the AI platform that generated the references during the verification phase. In the event of discrepancies regarding the classification of metadata accuracy or the assignment of the hallucination score, disagreements were resolved through discussion and consensus with a senior fourth researcher.

For each article that was confirmed to exist, official data were retrieved from scientific databases. The following variables were compiled in the study database and systematically compared with those originally provided by the AIs: title, authors, journal, year of publication, DOI, publication type, and total citation count.

In order to ensure strict methodological uniformity and mitigate bias in retrieving citation counts and classifying article types, a precise hierarchy of reference databases was established. The Scopus platform was used as the primary source and gold standard. Exclusively for articles not traceable in Scopus, the search for missing data was extended to Google Scholar and, ultimately, to ResearchGate.

The procedure for extracting official metadata was conducted entirely in April 2026; therefore, all time-dependent bibliometric parameters (particularly the number of citations) refer to this time frame.

#### Phase 3: Hallucination Score Calculation

In order to quantitatively assess the reliability of the bibliographic references and the incidence of “hallucinations,” an analytical scoring system was developed. This tool represents a methodological adaptation of the RHS, previously proposed in the literature for evaluating chatbots in the medical field [14,14].

This modification was necessary due to the different nature of the prompts used: while the original RHS protocol is designed for tasks where instructions regarding the metadata to be provided are already explicitly specified in the input, this study tested the models’ ability to autonomously generate bibliographies, starting from an open, generic request. As the AI was not required to provide specific data, the metric was simplified to a linear penalty scale that evaluates the model’s capacity to spontaneously generate the correct parameters required for source identification.

Specifically, the adopted scoring system assigns one penalty point for every inaccuracy detected during the fact-checking process (phase 2) against official databases.

The score for each article was determined using the following cumulative scheme: incorrect or incomplete title: +1; nonmatching or inaccurate author names: +1; incorrect journal (or publication source): +1; incorrect year of publication: +1; and invalid DOI or DOI not corresponding to the article: +1.

The total score for each reference, therefore, ranges from a minimum of 0 (exact match, no errors) to a maximum of 5 (errors across all parameters). In cases of “total hallucination”—that is, when the cited source proved to be entirely nonexistent, and it was impossible to trace any real article by cross-referencing the information generated by the AI—a maximum score of 5 points was assigned by default to indicate the complete unreliability of the generated resource.

### Statistical Analysis

Statistical analyses were conducted with the aim of describing the sample characteristics and comparatively evaluating the performance of the 4 selected AI models (Grok, ChatGPT, Meta AI, and Perplexity). The sample characteristics were summarized using descriptive statistics. The frequencies and percentages were used to describe categorically measured variables. Median values and IQR, alongside means and SDs, were used to describe quantitative and ordinal variables (latency times, number of citations, publication year, and hallucination score). For the analyses concerning publication year and citation count, only articles verified as existing following the fact-checking procedure were included; “total fabrications” (full hallucinations) were excluded from these computations, as they lack objective, verifiable metadata. The normality of the data distribution was verified using the Shapiro-Wilk tests. For the analysis of categorical variables (eg, the presence or absence of hallucinations across the different models), Pearson chi-square (*χ*²) test was applied. The nonparametric Kruskal-Wallis test was used to compare the models with respect to continuous and ordinal variables. For statistically significant findings, the analysis was further deepened using Dunn post hoc test, applying the Bonferroni correction for multiple comparisons. Furthermore, to account for potential data clustering and rule out any bias related to pseudoreplication (due to multiple references generated from a single prompt), a sensitivity analysis was conducted by aggregating the data and using the 36 independent query runs as the primary unit of analysis.

Data analysis was performed using R software (version 4.5.1; R Foundation for Statistical Computing). Specifically, data manipulation and descriptive statistics were conducted using the dplyr and gtsummary packages. The Shapiro-Wilk test, Pearson chi-square test, and the Kruskal-Wallis test were executed using the core stats package. Dunn’s post hoc pairwise comparisons were performed using the dunn.test package. The statistical significance level α was set at .05.

### Ethical Considerations

This study did not involve human participants, animal subjects, or personal patient data. The research consisted exclusively of interacting with publicly accessible AI platforms; therefore, institutional ethics committee approval was not required.

## Results

### Data Volume and General Overview

The data extraction process, which was conducted across 3 iterations for each of the 3 queries administered to the 4 AI platforms under examination, generated a theoretical pool of 360 bibliographic references (30 articles requested for each AI-query combination). However, the analysis of the generated output revealed a significant data dispersion compared to the expected volume. Progressive screening of the sample, as illustrated in Figure 1, yielded the distribution shown in Textbox 1.

**Figure 1.**
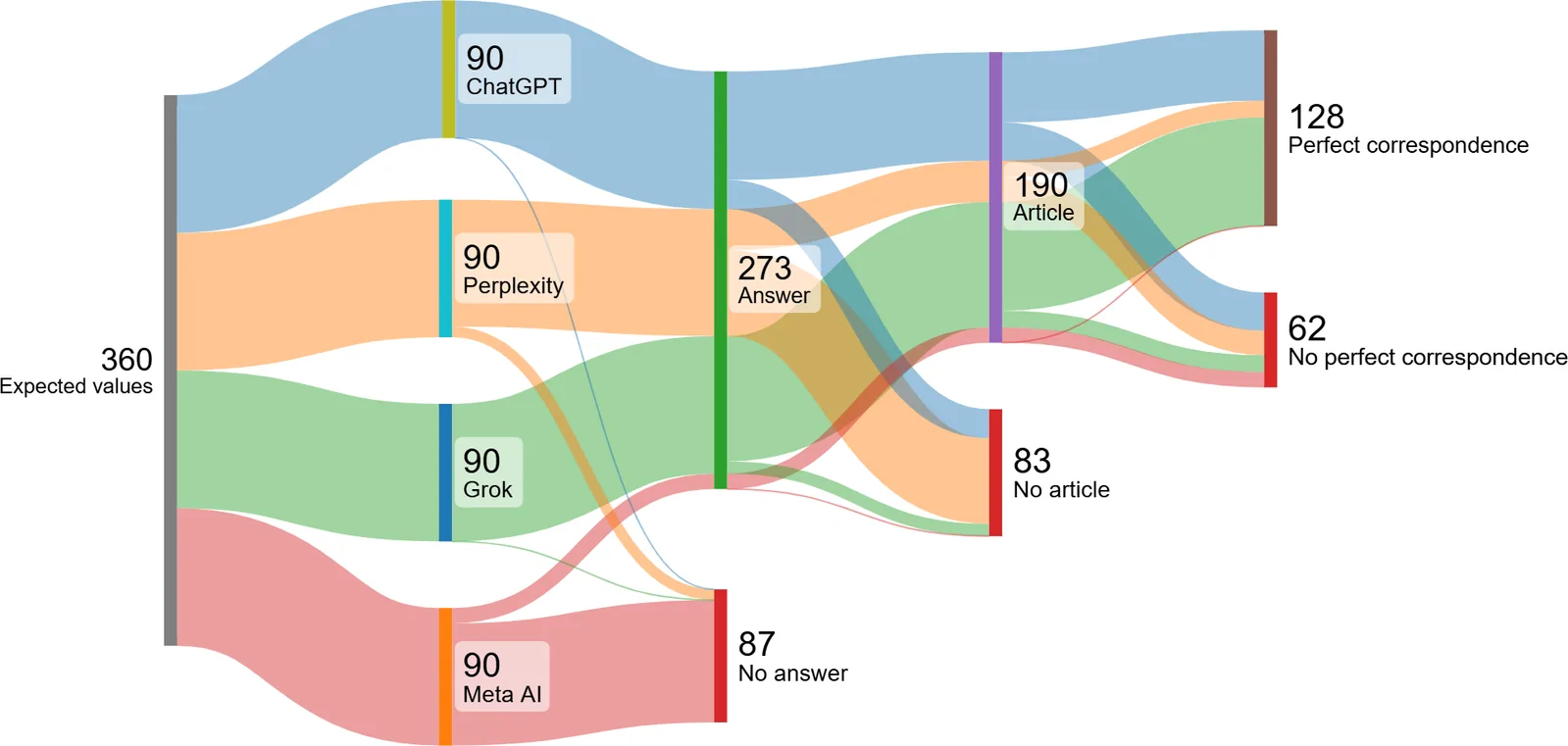
Sankey diagram illustrating the screening process and dispersion of the data sample. The diagram traces the path from the initial theoretical volume (n=360 references) to the 128 articles that were found to be accurate after the fact-checking process, highlighting the proportion of data lost due to system errors, ineligibility, and metadata hallucinations (diagram created with SankeyMATIC [20]).

Textbox 1. **Quantitative breakdown of the AI-generated responses and validation outcomes.**

- A total of 87 out of 360 (24.2%) null or error responses: A substantial portion of the queries failed to produce any bibliographic list and instead returned conversational messages or notifications indicating an inability to complete the task.
- A total of 273 out of 360 (75.8% of the theoretical total) generated responses: The models produced a total of 273 items. A qualitative analysis of this subset revealed broad typological heterogeneity, encompassing disparate sources such as books, websites, YouTube videos, guidelines, and blogs. Within this group, the data were further categorized as follows:
  - A total of 83 out of 360 “unacceptable” responses: Items not equivalent to peer-reviewed scientific articles, excluded during phase 1 due to noncompliance with the strict eligibility criterion.
  - A total of 190 out of 360 (52.8% of the theoretical total) articles: Items that strictly adhered to the requested “article” format and were subjected to the subsequent verification phase (phase 2). Following fact-checking against official databases, these 190 articles were further subdivided as follows:
    - A total of 62 out of 360 (17.2% of the theoretical total) articles with inaccuracies or hallucinations: Articles containing at least one error in the AI-provided metadata (title, authors, journal, year, or DOI not matching reality), thus incurring penalty points according to the hallucination score system.
    - A total of 128 out of 360 (35.6% of the theoretical total) articles with exact match: Only around a third of the initial theoretical sample passed all checks, demonstrating complete and exact correspondence and an absence of “hallucinations” in the analyzed metadata.

Building upon the data dispersion illustrated in Figure 1, the overall performance of the AI models can be stratified and evaluated across 3 distinct operational concepts, using the appropriate denominators for each specific phase:

1. Task completion (273/360 generated responses): The models successfully produced an output in 75.8% of the theoretical total, whereas 87 requests resulted in null or error messages.
2. Instruction adherence (190/273 valid formats): When the AI successfully generated a response, it adhered to the specific constraint of providing a peer-reviewed “article” format in 69.6% of the cases. The remaining 83 items were classified as unacceptable (eg, blogs, guidelines, and videos).
3. Metadata accuracy (128/190 exact matches): Focusing strictly on the 190 correctly formatted articles, the fact-checking process verified that 67.4% of them were completely accurate and free of hallucinations. The remaining 62 (32.6%) articles contained at least one generated inaccuracy.

### Analysis of Response Times (Latency)

The analysis of response times, calculated from the moment of prompt submission to the completion of the output, revealed performance differences among the analyzed platforms.

ChatGPT demonstrated the highest overall consistency, maintaining brief processing times across all iterations, ranging from a minimum of 8 to a maximum of 17 seconds, with median values of 15.0 (IQR 9‐15) and mean values of 13.2 (SD 3.8) seconds.

In contrast, Grok required systematically longer processing cycles compared to the other models. Its processing times consistently remained above 28 seconds, with a median time of 47.0 (IQR 39.0‐66.0) seconds and a maximum peak of 97 seconds.

Meta AI exhibited highly fluctuating trends, resulting in an overall median of 15.0 (IQR 9.0‐25.7) seconds and a mean of 18.8 (SD 11.4) seconds. Although it recorded the shortest response time of the entire experiment (6 s in the D3-R1 trial, coinciding with the delivery of an error message), it experienced significant slowdowns during other queries, reaching peaks of up to 40 seconds.

Perplexity emerged as the fastest model, with an average processing time of 12.3 seconds and a median of 10.0 (IQR 8.0‐15.0) seconds.

### Analysis of Query D1: Aflatoxin Toxicity

The first query revealed diametrically opposed reliability rates among the models:

1. ChatGPT demonstrated high formal consistency, returning 27 valid articles out of 30 (90%) across the 3 iterations. Of these, 21 out of 27 (77.8%) achieved an exact match (perfect metadata). The incidence of hallucinations remained limited, totaling a cumulative hallucination score of 6. The proposed sources showed a very high bibliometric impact: median citations 251.0 (IQR 37.5‐814.0), with a median publication year of 2020.0 (IQR 2010.5-2023.0; mean 2017.1, SD 7.6).
2. Grok recorded maximum adherence to the prompt, providing 30 valid articles out of 30 (100%), of which 22 had exact matches, resulting in a total hallucination score of 12. The suggested literature was more recent (median year 2022.0, IQR 2019.0-2023.8) with a median of 111.5 (IQR 21.3‐393.5) citations.
3. Meta AI consistently failed the task. Across all 3 iterations, it produced conversational outputs or error messages.
4. Perplexity exhibited drastic internal instability. It only produced 9 valid articles in the second iteration R2 (of which 4 were exact matches, with a total hallucination score of 18); in the other 2 trials (R1 and R3), it did not generate any acceptable references, concluding with a total of 9 valid articles out of 30.

### Analysis of Query D2: Hospital Environment Sanitization

Query D2 severely challenged the models’ ability to discern “scientific articles” from other types of institutional documents (such as guidelines or hospital reports).

1. ChatGPT yielded fewer valid articles, returning only 16 out of 30 (53.3%). However, the valid sources maintained good reliability: 12 references achieved an exact match, generating a cumulative hallucination score of 9. The bibliometric impact recorded a median value of 10.5 (IQR 5.0‐39.5) citations and a median publication year of 2021.0 (IQR 2019.0-2023.5).
2. Grok, despite providing fewer valid articles than usual (22 out of 30, equating to 73.3%), recorded its highest precision peak: 20 out of the 22 references achieved an exact match, limiting the total hallucination score to a value of 2. The suggested literature showed a median value of 36.5 (IQR 17.3‐126.3) citations and a median publication year of 2021.5 (IQR 2018.0-2023.0).
3. Meta AI failed to complete the task. Across all 3 iterations, it produced conversational outputs or error messages.
4. Perplexity demonstrated an inability to adhere to the formal constraints of the prompt, returning zero valid articles out of 30 across all iterations and frequently substituting the requested bibliographic list with narrative summaries or general guidelines.

### Analysis of Query D3: Human Papilloma Virus Vaccination

The query regarding a topic of broad public health relevance revealed the concerning phenomenon of “hallucinatory drift,” which is characterized by a drop in consistency during serial repetitions of the same prompt.

1. ChatGPT, despite generating 28 valid articles out of 30 (93.3%), suffered a dramatic drop in intra-prompt reliability. While in the first 2 trials (R1, R2) there were a total of 12 exact matches, in the third iteration (R3), only one was produced. This collapse is reflected in the hallucination score, which reached a total of 55 points, 45 of which were concentrated exclusively in R3, indicating an almost total fabrication of sources in the final trial. Regarding bibliometric characteristics, the suggested literature recorded a median value of 168.0 (IQR 20.5‐1093.5) citations and a median publication year of 2020.0 (IQR 2015.0-2022.0).
2. Grok recorded its best overall performance on this query. It returned 30 valid articles out of 30 (100%), with 96.7% (29/30) exact matches. The cumulative hallucination score was the lowest of the entire experiment, limited to just 1 penalty point. The bibliometric impact showed a median value of 1.0 (IQR 0.0‐32.0) citations, alongside a median publication year of 2025.0 (IQR 2020.0-2026.0). Notably, in trial D3-R3, the algorithm extracted and returned only literature produced in the current year (2026), confirming a strong inclination toward up-to-date sources.
3. Meta AI generated data solely in the second iteration (providing 10 valid articles in R2) and systematically failed in subsequent trials. However, the fact-checking analysis conducted on this single trial revealed zero exact matches and a critical hallucination score of 30, translating to an alarming mean value of 3.0 (SD 1.8) errors for each proposed article.
4. Perplexity provided 18 valid articles out of 30 (extracted between R2 and R3). However, metadata precision was very poor: only 7 exact matches against a total hallucination score of 32 (9 in R2 and 23 in R3). The bibliometric data revealed a median publication year of 2011.0 (IQR 2007.0-2019.0) and an extremely high median citation count of 1159.0 (IQR 927.0‐1470.0).

### Comparative Performance Analysis: Individual Model Behavior

### Overall Performance Metrics

#### Overview

The data collected provides a multifaceted overview of the actual capabilities of each model, highlighting profoundly different approaches to managing bibliographic data retrieval and source selection strategies. Table 1 summarizes the descriptive statistics for all parameters evaluated across the 4 platforms.

Figure 2 illustrates the results regarding the effectiveness of each model in providing valid responses (free from systematic errors or complete generation failures), offering an initial qualitative assessment of the generated outputs.

**Table 1.** Descriptive analysis of bibliographic sources and hallucinations across AI models^a^.

AI model/parameter	Sample size (n)^b^	Median (IQR)	Mean (SD; range)
ChatGPT			
Hallucination score (0‐5)^c^	71	0.0 (0.0‐1.0)	1.0 (1.7; 0-5)
Publication year	61	2020.0 (2011.0‐2023.0)	2017.7 (7.0; 2003-2025)
Citation count	60	73.5 (10.8‐718.0)	419.5 (601.2; 0-2640)
Grok			
Hallucination score (0‐5)^c^	82	0.0 (0.0‐0.0)	0.2 (0.5; 0-2)
Publication year	82	2022.0 (2019.0‐2024.8)	2020.9 (4.9; 2004-2026)
Citation count	82	32.0 (2.5‐148.3)	141.8 (265.8; 0-1480)
Meta AI			
Hallucination score (0‐5)^c^	10	2.5 (1.3‐5.0)	3.0 (1.8; 1-5)
Publication year	6	2014.5 (2008.0‐2018.8)	2013.7 (6.1; 2007-2025)
Citation count	6	1068.0 (513.0‐1450.5)	1016.7 (664.0; 168-1890)
Perplexity			
Hallucination score (0‐5)^c^	27	1.0 (0.0‐5.0)	1.9 (2.2; 0-5)
Publication year	19	2009.0 (2006.5‐2017.5)	2010.8 (6.6; 2001-2021)
Citation count	19	999.0 (730.5‐1339.5)	1012.7 (466.3; 143-1890)

a This table presents a comparative descriptive analysis focusing on 2 macro-dimensions: the formal characterization of real identified bibliographic sources (expressed via publication year and citation count) and the quantitative incidence of metadata errors (measured via the hallucination score on a 0‐5 scale).

b For the analysis of publication year and citation count, the sample size decreases significantly for Meta AI (n=6) and Perplexity (n=19) because it excludes “total fabrications” (references entirely invented by the models). Being completely nonexistent, these fabricated sources cannot be traced in official scientific databases and thus lack objective chronological and citation metadata. The minor discrepancy for ChatGPT citations (n=60) is attributed to missing bibliometric data on academic databases.

c Sample sizes vary across parameters due to the probabilistic nature of the generated outputs. For the “Hallucination Score,” the sample includes all scientific references initially produced by the platforms (71 for ChatGPT, 82 for Grok, 10 for Meta AI, and 27 for Perplexity).

**Figure 2.**
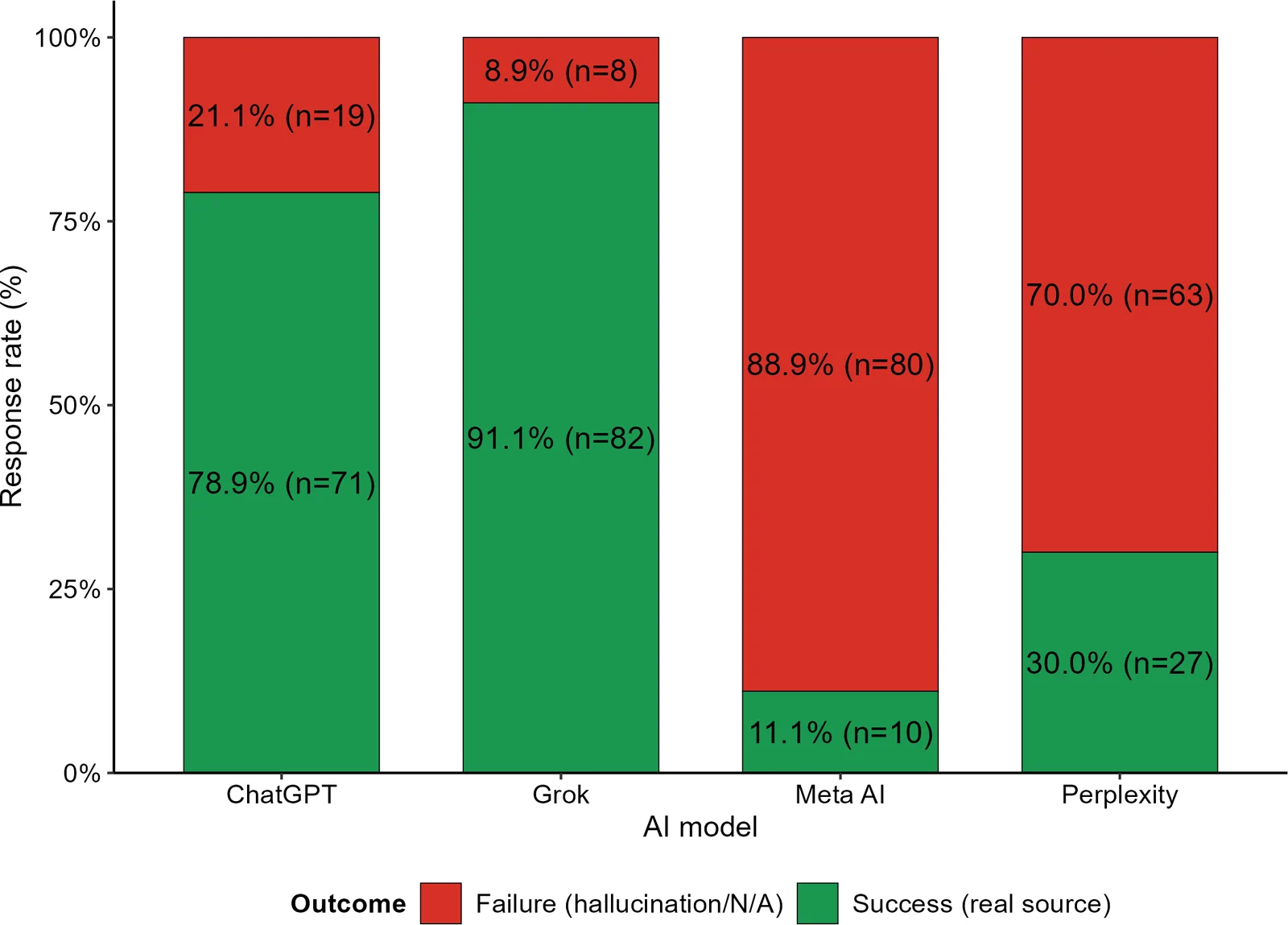
Output reliability analysis and success rates in retrieving valid sources for ChatGPT, Grok, Meta AI, and Perplexity. The chart was generated using R software (version 4.5.1). N/A: not applicable.

Following the evaluation of the AI models’ ability to generate valid responses, the study focused on the accuracy of the real sources identified. Initially, this analysis was conducted on a categorical basis across 3 distinct outcome levels: exact match (completely accurate reference), incorrect match (presence of hallucinations or metadata inaccuracies), and null response (failure to generate an output or nonarticle format). The association analysis, performed using Pearson chi-square test, confirmed that the ability to provide real references differs significantly depending on the specific platform used (*χ*_6_²=192.5; *P*<.001).

To clarify the distribution of these outcomes and present the exact data matrix used for the chi-square analysis, the complete frequencies for each platform are detailed in Table 2.

To investigate the nature of these statistical discrepancies, the analysis was extended to the analytical distribution of the hallucination score scale. Figure 3 presents the frequency map of penalty scores for each model, allowing the recurrence of specific inaccuracies and the propensity of each AI toward partial hallucinations or complete metadata fabrications.

**Table 2.** Contingency table of reference generation outcomes by AI platform.

Platform	Exact match^a^, n	Incorrect match^b^, n	Null response^c^, n	Total
ChatGPT	46	25	19	90
Grok	71	11	8	90
Meta AI	0	10	80	90
Perplexity	11	16	63	90
Total	128	62	170	360

a “Exact Match” indicates perfectly accurate and verifiable references.

b “Incorrect Match” comprises references containing any degree of hallucination or metadata inaccuracy.

c “Null Response” encompasses unanswered requests, system error messages, refusals to execute the prompt, and outputs that failed to comply with the required peer-reviewed article format (eg, blogs, websites, and guidelines).

**Figure 3.**
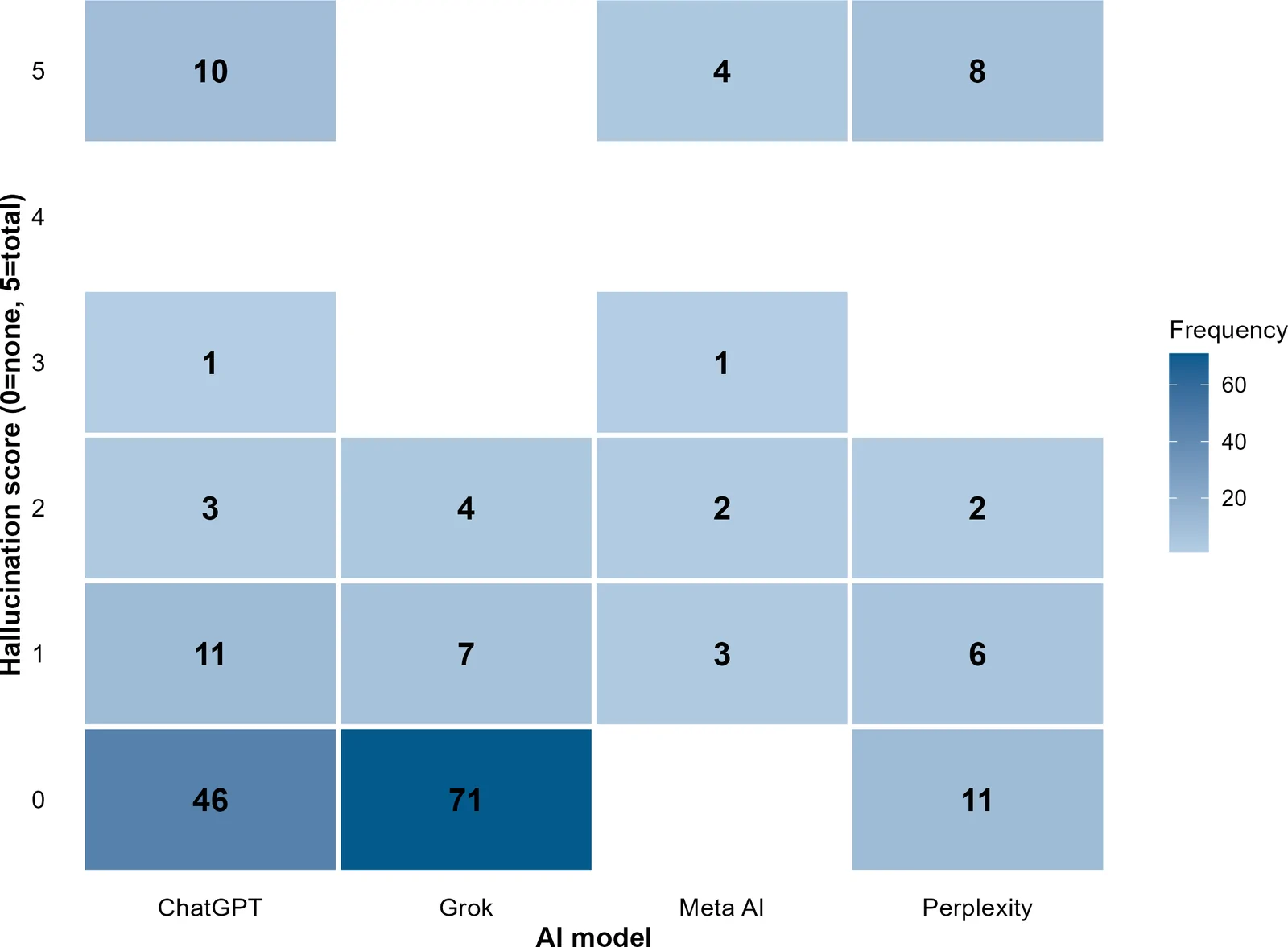
Hallucination score frequency map. The heatmap illustrates the frequency distribution of penalty scores (ranging from 0 to 5) for each examined AI model. Graph generated using R software (version 4.5.1).

#### ChatGPT

The model demonstrated robust formal stability, completing all 90 requested references and applying a good initial filter for prompt adherence (71/90, 78.9% real articles). Accuracy proved to be moderate, yielding 46 exact matches and an average hallucination rate of 0.97 per response. An analysis of the selection logic shows that the model tends to rely on modern yet consolidated literature, with a median publication year of 2020.0 (IQR 2011.0-2023.0) and a median academic impact of 73.5 (IQR 10.8‐718.0) citations.

#### Grok

This model distinguished itself as the most accurate and adherent to the rigorous demands of medical research. It successfully completed the entire task (90/90 requested references), ensuring an excellent formal filter (82/90, 91.9% valid articles) and exceptional factual reliability, yielding 71 exact matches. Its hallucination rate was minimal (mean of 0.2, SD 0.5). However, the most innovative aspect concerns its information retrieval criteria: Grok clearly prioritizes the recency of source (most recent median publication year of 2022.0, IQR 2019.0-2024.8), presenting a lower median number of citations (32.0, IQR 2.5‐148.3) compared to its competitors. This strong preference for up-to-date literature found its fullest expression in the D3-R3 iteration, in which the algorithm exclusively extracted and retrieved articles published in the current year (2026). This behavior highlights a marked tendency in its output to prioritize recently published literature over older, highly cited sources.

#### Meta AI

Meta AI proved to be the least effective platform for this specific academic task. The model only provided a response consistent with the prompt on one occasion and generated a total of just 10 articles. None of these passed the fact-checking screening (zero exact matches). It accumulated a total hallucination score of 30 in the single provided response (D3-R2), translating to an average of 3 penalty points per reference. The few real sources identified (n=6) were dated (median 2014.5, IQR 2008.0-2018.8) but possessed an extremely high bibliometric impact (median 1068.0, IQR 513.0‐1450.5 citations). Meta AI thus demonstrated a marked tendency to fabricate metadata entirely, revealing the inadequacy of its current architecture in responding to structured clinical queries.

#### Perplexity

Although the platform guaranteed a high output volume (returning 83 references out of the 90 requested), it exhibited a weak formal filter: only 27 items proved to be scientific articles, while the remainder belonged to “gray literature” (YouTube videos, noninstitutional blogs, dissertations, etc). The fact-checking conducted on these 27 articles revealed that 8 (29.6%) of them were total fabrications, rendering the retrieval of real bibliographic metadata impossible. Consequently, while the hallucination score was calculated for all the generated articles (n=27), analyses of publication year and citation count were conducted on the 19 articles that could be verified as existing. Within this subset, 11 exact matches were recorded, with a mean hallucination rate of 1.9 (SD 2.2). The low median publication year (median 2009, IQR 2006.5-2017.5) and the high citation count (median 999.0, IQR 730.5‐1339.5) indicate that, in this test, the platform predominantly retrieved older, highly cited documents and non-peer-reviewed gray literature, rather than recent, specific scientific articles (Figure 4).

**Figure 4.**
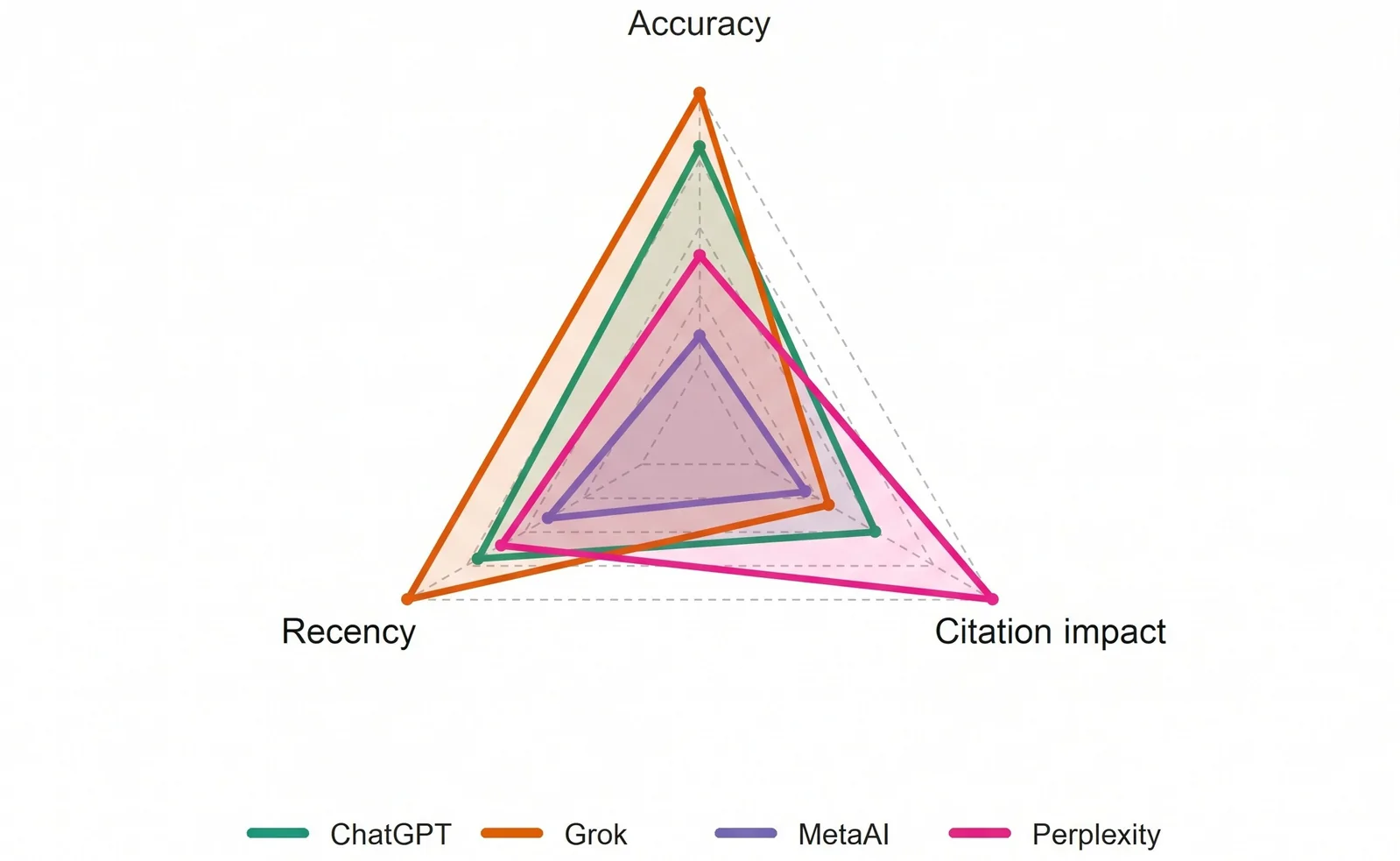
AI model performance profiles. The radar chart illustrates a multivariate comparison of the 4 generative AI platforms across 3 key dimensions: accuracy (absence of hallucinations), citation impact (volume of citations of the retrieved sources), and recency (propensity to select recently published literature). Values are normalized on a 0‐10 scale, where the outer vertex represents maximum performance. Graph generated using R software (version 4.5.1).

### Inferential Analysis and Statistical Significance

To scientifically validate the observed divergences among the models, an inferential analysis was conducted on a total sample of 360 observations. Shapiro-Wilk normality tests yielded extremely low *P* values for all variables (*P*<.001), confirming a nonparametric data distribution and justifying the use of the Kruskal-Wallis test.

The Kruskal-Wallis test revealed statistically significant differences among the 4 models across all analyzed dimensions (*P*<.001 in all tests).

Pairwise comparisons with Bonferroni correction allowed for the mapping of statistical distances between the platforms:

1. Hallucination score: Grok proved to be statistically more reliable than ChatGPT (*P*=.007) and demonstrated a clear superiority over Meta AI (*P*<.001) and Perplexity (*P*<.001). Furthermore, ChatGPT maintained a significantly lower error profile compared to Meta AI (*P*<.001).
2. Source chronology (year of publication): Grok demonstrated a statistically superior ability to retrieve recent sources compared to Perplexity (*P*<.001), Meta AI (*P*=.01), and ChatGPT (*P*=.02). ChatGPT also proved to be more focused on recent literature than Perplexity (*P*=.001).
3. Citation count: The volume of citations retrieved by Perplexity and Meta AI was significantly higher than that retrieved by both Grok (*P*<.001 and *P*<.001, respectively) and ChatGPT (*P*<.001 and *P*=.03, respectively). In turn, ChatGPT extracted significantly more cited sources than Grok (*P*=.03). However, the combined analysis indicates that this superiority in citation volume for Meta AI and Perplexity does not reflect a greater overall authority of the models. Rather, it is based on an extremely reduced sample of valid articles (n=6 for Meta AI and n=19 for Perplexity), juxtaposed against a systematic failure to provide compliant outputs or the total fabrication of sources for the rest of the prompts (Figure 5).

To ensure the robustness of our findings and strictly account for data clustering, we reevaluated the data using the 36 independent query runs as the primary unit of analysis (n=36). The Kruskal-Wallis test on this aggregated dataset confirmed the original findings, demonstrating significant performance disparities among the four platforms (*P*=.004 for the median hallucination score, and *P*<.001 for the percentage of exact matches). This confirms that the observed differences in AI model accuracy are inherent to the platforms’ architectures and not an artifact of pseudoreplication.

**Figure 5.**
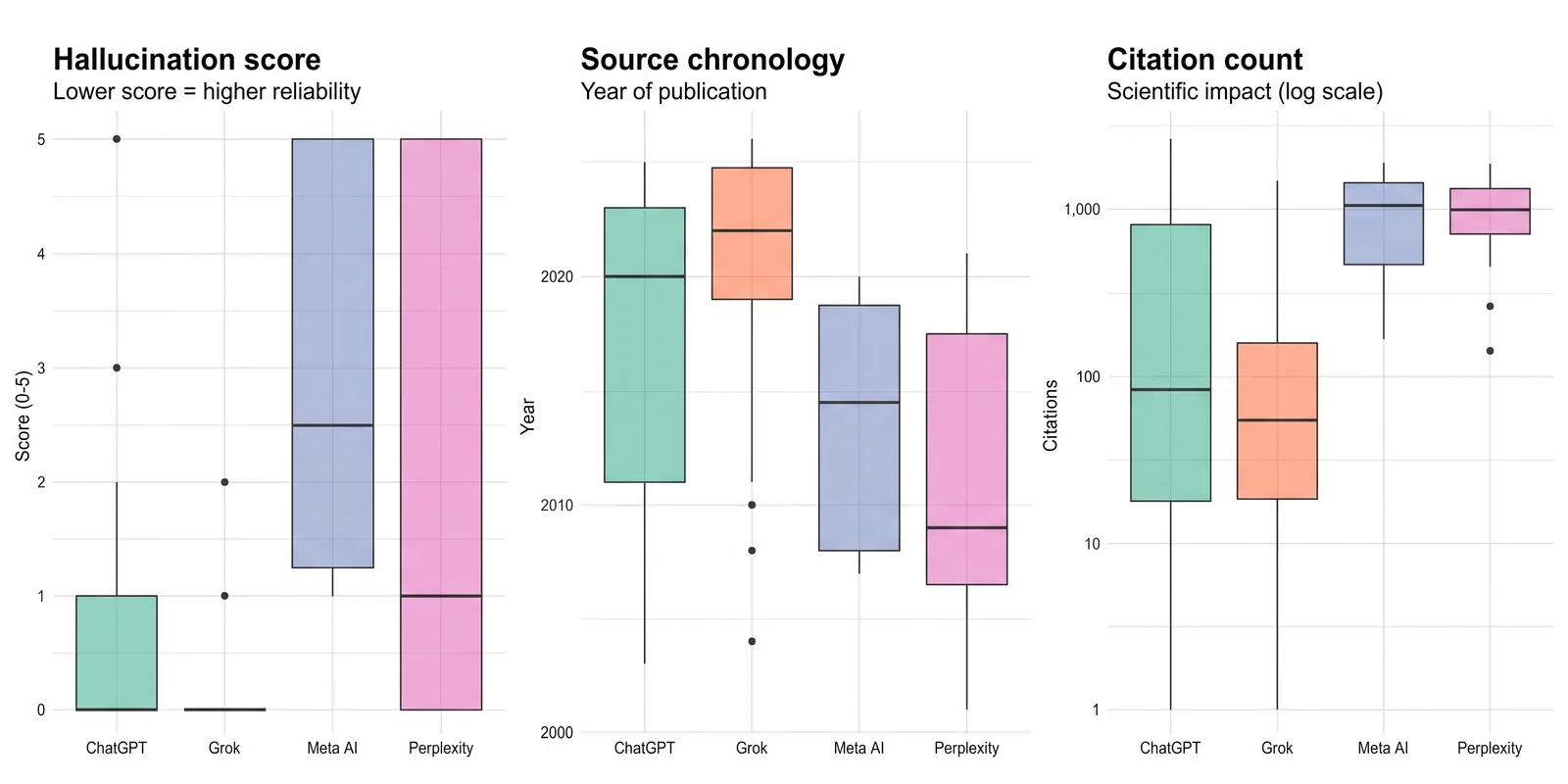
Comparative performance analysis of the AI models. From left to right: hallucination score (error distribution), source chronology (source publication year), and citation count (bibliometric impact on a logarithmic scale). Graph generated using R software (version 4.5.1).

## Discussion

### Principal Findings

The findings reveal a highly critical scenario: despite the remarkable linguistic fluency and the apparent confidence with which most models generate their responses, the actual factual accuracy of the metadata is alarming. The most significant finding of our investigation lies in the stratified breakdown of the models’ failures. While the overall strict yield was only 35.6% (128/360), a deeper analysis reveals distinct operational bottlenecks: the models failed to complete the task in nearly a quarter of cases, struggled with instruction adherence (generating nonarticle sources 30.4% of the time), and, most critically, hallucinated metadata in 32.6% (62/190) of the formally correct articles. A large proportion of the output was divided between unacceptable formats (gray literature, videos, and blogs) and articles containing at least one inaccuracy (62/360, 17.2%).

In line with recent findings in the literature [9], our data confirm that the use of the term “hallucination” risks downplaying the severity of the phenomenon in a clinical context: the analyzed chatbots engaged in outright data fabrication, generating nonexistent DOI codes or combining real authors with the titles of articles that were never published. This behavior, inherent to the probabilistic nature of LLMs, poses a severe risk to the integrity of EBM, exposing both health care professionals and patients to the dangers of scientific misinformation.

### Practical Implications

The findings of this study have significant implications for various categories of users. For health care professionals, the uncritical use of LLMs for clinical updates or evidence-based research poses an ethical and legal risk: basing a therapeutic or diagnostic decision on “fabricated” references or incorrect metadata can compromise patient safety and the effectiveness of treatment. In the academic setting, for students and researchers, relying on such tools without meticulous manual verification of sources threatens scientific integrity, exposing them to the risk of unintentional plagiarism or the propagation of nonexistent citations. Finally, for the general public, the apparent authority with which chatbots cite (often nonexistent) medical studies fuels the phenomenon of health misinformation, creating a false perception of scientific rigor that may lead to dangerous self-diagnosis. In summary, the results confirm that, at present, generative AI platforms cannot be considered substitutes for validated bibliographic databases (such as PubMed or Scopus). Instead, they must be regarded exclusively as complementary support tools, subject to constant and rigorous fact-checking by domain experts.

### Comparison With Prior Work

The urgency to address these practical implications is further substantiated by recent scientific evidence. Indeed, the reliability of the results emerging from our study is, in fact, significantly corroborated by a recent analysis conducted by Cabezas-Clavijo and Sidorenko Bautista [21]. Although the authors investigated a different panel of platforms, their research arrived at the same structural conclusions. Specifically, even across varying algorithmic architectures, their study confirmed that none of the tested artificial intelligence tools is currently capable of ensuring absolute precision, reiterating the inadequacy of these models to serve as primary, error-free academic search engines. This convergence demonstrates that the tendency toward bibliographic falsification is not limited solely to the platforms we analyzed but represents a systemic limitation of current generative technology.

### Study Limitations

Despite the methodological rigor applied, this study is not without its limitations. First, although the analyzed prompts were subjected to triple iteration to test for stability, the sample size is limited compared to the infinite variety of potential clinical queries. Future studies using larger datasets could provide a more comprehensive analytical overview. Second, the rapid technological evolution characterizing LLMs must be considered. As the tested models are updated frequently, the observed performances represent a snapshot of the current technological landscape, which is subject to rapid change.

A further limitation concerns the language used and the way in which the queries were posed to the AI. Since these systems are predominantly trained on English-language text, prompting in Italian may have partially compromised their ability to retrieve international scientific literature accurately.

Furthermore, the decision to use direct queries devoid of contextual instructions on how to structure the output—simulating the behavior of an average user—precluded the opportunity to assess whether more detailed prompt engineering would have guided the models toward more adequate and rigorous responses.

A further significant limitation concerns the selection of the 3 query topics. The topics were chosen subjectively to represent a gradient of specificity, and only a single prompt formulation was used for each domain. Based on the current study design, it is not possible to definitively determine whether the observed performance variations across the three queries were driven by the intended level of specificity, the sheer volume of available literature in each respective field, potential nuances in terminology translation, varying coverage within the training databases, or the intrinsic architectural performances of the AI models. Future studies should employ multiple, validated prompts across standardized topic clusters to isolate these variables more effectively.

Finally, it is important to emphasize that this study analyzed only the free, base versions of the four platforms. Although this methodological choice accurately reflects the tools accessible to the majority of everyday users (eg, students, laypersons, or professionals without paid subscriptions), it prevents the evaluation of these systems’ maximum technological capabilities. Premium versions incorporate higher-performing models and advanced web search capabilities, which could mitigate the tendency to generate fictitious bibliographies.

### Conclusions

This study demonstrates that, under the specific testing conditions evaluated, the selective generative AI platforms lack the structural reliability required to replace traditional bibliographic databases in medical and scientific research. Although the conversational interfaces provide a highly intuitive user experience, the alarming rate of hallucinations poses a significant threat to the integrity of EBM.

Specifically, the comparative analysis revealed significant operational differences between the tools tested. While models such as Meta AI and Perplexity proved entirely inadequate for the rigor demanded by the academic setting, ChatGPT demonstrated greater formal stability, anchoring itself to historically established evidence with high bibliometric impact (retrieving, on average, highly cited sources). Conversely, within the confines of our sample, Grok exhibited the best overall performance: although it retrieved articles with lower historical citation volumes, it distinguished itself through high factual accuracy and a pronounced ability to monitor contemporary literature. However, none of the examined platforms proved entirely immune to generating errors or inventing sources, confirming that the risk of data fabrication is currently a limitation of the model versions and configurations tested.

In conclusion, the integration of generative AI into clinical and academic practice should not be impeded; rather, it demands an urgent and profound shift in methodology. As advocated in recent literature, the introduction of structured AI literacy programs [22] throughout the entire educational pathway [23] has become essential: starting from school curricula [24] through university education (especially for medical and scientific degree courses) to continuous medical education programs. In a high-risk sector such as health care, these systems must be framed as complementary exploratory tools rather than primary search engines. The professional’s critical appraisal and rigorous verification against official databases remain indispensable, ethically obligatory steps that cannot be delegated to a machine. Only through formal education regarding the limitations and probabilistic mechanisms of LLMs will it be possible to harness the potential of these technologies without compromising patient safety and the authority of scientific research.

## Abbreviations

EBM: evidence-based medicine
LLM: large language model
NLP: natural language processing
RHS: reference hallucination score

## References

[1] (2018). Artificial intelligence (AI) in healthcare and research. https://www.nuffieldbioethics.org/wp-content/uploads/Artificial-Intelligence-AI-in-healthcare-and-research-1.pdf.

[2] Mansoor I, Abdullah M, Rizwan MD, Fraz MM (2026). Reasoning with large language models in medicine: a systematic review of techniques, challenges and clinical integration. Health Inf Sci Syst.

[3] Wang X, He X, Wei J, Liu J, Li Y, Liu X (2023). Application of artificial intelligence to the public health education. Front Public Health.

[4] Sarker IH (2024). LLM potentiality and awareness: a position paper from the perspective of trustworthy and responsible AI modeling. Discov Artif Intell.

[5] Lee H (2024). The rise of ChatGPT: exploring its potential in medical education. Anat Sci Educ.

[6] Wei Q, Yao Z, Cui Y, Wei B, Jin Z, Xu X (2024). Evaluation of ChatGPT-generated medical responses: a systematic review and meta-analysis. J Biomed Inform.

[7] Zhou M, Gui F, Ai C (2026). Comparing the performance of four mainstream large language models on medical literature review generation: a human expert evaluation in SMILE surgery. Graefes Arch Clin Exp Ophthalmol.

[8] Ji Z, Lee N, Frieske R (2023). Survey of hallucination in natural language generation. ACM Comput Surv.

[9] Emsley R (2023). ChatGPT: these are not hallucinations - they’re fabrications and falsifications. Schizophrenia.

[10] Ahmad M, Yaramic I, Roy TD (2023). Creating trustworthy LLMs: dealing with hallucinations in healthcare AI. arXiv.

[11] Walters WH, Wilder EI (2023). Fabrication and errors in the bibliographic citations generated by ChatGPT. Sci Rep.

[12] Bhattacharyya M, Miller VM, Bhattacharyya D, Miller LE (2023). High rates of fabricated and inaccurate references in ChatGPT-generated medical content. Cureus.

[13] Goodman RS, Patrinely JR, Stone CA (2023). Accuracy and reliability of chatbot responses to physician questions. JAMA Netw Open.

[14] Aljamaan F, Temsah MH, Altamimi I (2024). Reference hallucination score for medical artificial intelligence chatbots: development and usability study. JMIR Med Inform.

[15] ChatGPT.

[16] Grok.

[17] Meta AI.

[18] Perplexity AI.

[19] OpenEvidence.

[20] SankeyMATIC.

[21] Cabezas-Clavijo Á, Sidorenko-Bautista P (2026). Assessing the performance of 8 AI chatbots in bibliographic reference retrieval: Grok and DeepSeek outperform ChatGPT, but none are entirely accurate. J Data Info Sci.

[22] Yi Y (2021). Establishing the concept of AI literacy. Eur J Bioeth.

[23] Kong SC, Korte SM, Burton S (2025). Artificial intelligence (AI) literacy – an argument for AI literacy in education. Innov Educ Teach Int.

[24] Ng DTK, Su J, Leung JKL, Chu SKW (2024). Artificial intelligence (AI) literacy education in secondary schools: a review. Interact Learn Environ.

